# On epigenetic stochasticity, entropy and cancer risk

**DOI:** 10.1098/rstb.2023.0054

**Published:** 2024-04-22

**Authors:** Andrew E. Teschendorff

**Affiliations:** CAS Key Laboratory of Computational Biology, Shanghai Institute of Nutrition and Health, Shanghai Institute for Biological Sciences, University of Chinese Academy of Sciences, Chinese Academy of Sciences, 320 Yue Yang Road, Shanghai 200031, People's Republic of China

**Keywords:** DNA methylation, stochasticity, cancer risk, ageing, epigenetic clock

## Abstract

Epigenetic changes are known to accrue in normal cells as a result of ageing and cumulative exposure to cancer risk factors. Increasing evidence points towards age-related epigenetic changes being acquired in a quasi-stochastic manner, and that they may play a causal role in cancer development. Here, I describe the quasi-stochastic nature of DNA methylation (DNAm) changes in ageing cells as well as in normal cells at risk of neoplastic transformation, discussing the implications of this stochasticity for developing cancer risk prediction strategies, and in particular, how it may require a conceptual paradigm shift in how we select cancer risk markers. I also describe the mounting evidence that a significant proportion of DNAm changes in ageing and cancer development are related to cell proliferation, reflecting tissue-turnover and the opportunity this offers for predicting cancer risk via the development of epigenetic mitotic-like clocks. Finally, I describe how age-associated DNAm changes may be causally implicated in cancer development via an irreversible suppression of tissue-specific transcription factors that increases epigenetic and transcriptomic entropy, promoting a more plastic yet aberrant cancer stem-cell state.

This article is part of a discussion meeting issue ‘Causes and consequences of stochastic processes in development and disease’.

## 1. Background

The word ‘stochastic’ derives from the ancient Greek *στ*ό*χο*ς (stókhos) meaning ‘to take a guess’. In biological sciences it is often used to refer to biological processes that by and large appear random. One of these biological processes that has gained significant attention from the community has been the gradual age-associated accumulation of molecular alterations in the normal cells of our bodies, which leads to cellular dysfunction and ageing [1–3]. While the main focus has been on alterations to DNA, notably somatic mutations [1], over the last decades there has been an increased interest in epigenetic alterations, including those which involve a covalent modification of DNA, known as DNA methylation (DNAm) [4–6].

DNAm refers to the covalent attachment of a methyl CH_3_ group to cytosines, usually, but not exclusively, in a CG context (denoted ‘CpG’ because of the phosphodiester bond) [7]. For any given CpG site in any given cell, DNAm is effectively binary (0 = both alleles unmethylated, 1 = both alleles methylated), except for a small number of loci that display allele-specific methylation (one allele methylated, the other unmethylated). According to most recent estimates, there are about 32 million CpG sites in the human genome [8,9], with the methylation state of each CpG largely determined by its local CpG density [10]. In particular, the great majority of CpGs are relatively isolated mapping to low CpG-dense regions that are generally methylated, while the smaller proportion of unmethylated CpGs tend to cluster together in high CpG-dense regions known as CpG islands (CGIs). Thus CpG density determines to a large extent the direction in which DNAm changes are acquired, with CGIs and low CpG-dense regions generally gaining and losing DNAm, respectively. CGIs co-localize with approximately 60–70% of gene promoters [11], with the propensity of these promoters to gain DNAm also influenced by the level of CpG density, with those of intermediate density more likely to display DNAm increases compared to promoters with highest CpG density [12,13]. Of note, DNAm differences between cell-types are not only restricted to gene promoters, but also to the lower CpG-dense regions that surround CGIs, termed ‘shores’ [14,15]. DNAm in regulatory regions such as promoters and enhancers is associated with gene expression, this association being nonlinear: on average, 70% of methylated gene promoters are associated with silencing of the corresponding gene [16], while unmethylated promoters only signal a ‘permission to express' and hence are largely uninformative. Thus, if DNAm changes are acquired in the normal cells of a tissue, such changes could be associated with corresponding changes in nearby gene expression, which in turn could affect a cell's function [17]. Current evidence points towards such DNAm changes being both causal for, as well as a consequence of, changes in nearby gene expression [18]. For instance, DNAm in a gene-promoter can act as a barrier preventing binding of a transcription factor (TF), leading to dysregulation of its gene-target [18]. Alternatively, it has been shown how reduced expression of a TF, for instance as mediated by a single nucleotide polymorphism, can lead to genome-wide increased DNAm levels at its binding sites [19].

## 2. Quasi-stochastic accrual of DNA methylation changes with age

**Figure 1.**
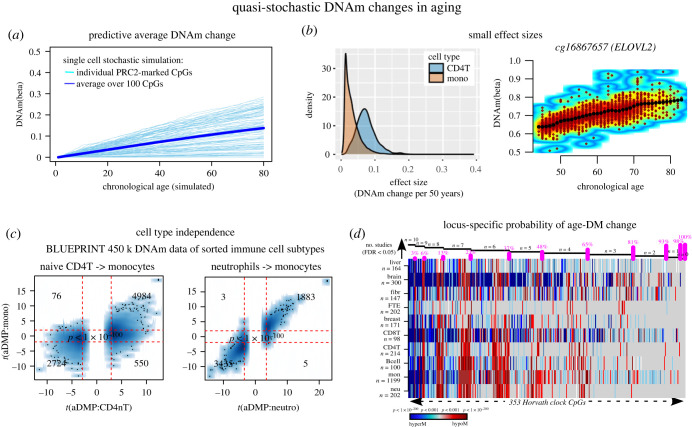
Quasi-stochastic DNAm changes in ageing. (*a*) Cumulative average DNAm over a pool of 1000 stem cells and 100 CpGs as a function of chronological age for one random tissue and subject. Data derives from a simulation model at single-cell resolution where each CpG has a distinct probability to acquire DNAm with probabilities of DNAm change per year ranging from 0 to 0.004. The average is shown in blue, the patterns of DNAm accrual for each CpG are shown in skyblue. (*b*) Left panel: effect size distribution for significant age-DMCs as derived from an Illumina 450k dataset profiling 1002 monocytes and 214 naive T-cells. Effect size is defined by the absolute change in DNAm over a 50 year period, with DNAm defined on a scale between 0 and 1. Right panel: density heatmap scatterplot of DNAm as a function of age for the top-ranked CpG in monocytes. Black dots are derived from a loess fit. (*c*) Scatterplot of *t*-statistics of age-association as derived in one immune cell subtype against the corresponding statistics in another immune cell-type. DNAm 450k data derives from BLUEPRINT. Panel adapted from Zhu *et al.* [31]. (*d*) Heatmap of age-associations for the 353 Horvath clock CpGs ranked by the number of tissues/cell types where the CpG is significantly changing in. Colour indicates directionality of change (blue = hyperM, red = hypoM).

One of the first studies to explore age-associated DNAm changes over multiple loci was the study by Fraga *et al.* [20], which compared DNAm profiles in lymphocytes of monozygotic twin pairs as a function of chronological age. This study observed that the intra-pair discordance of DNAm increased with the age of the twin pair, and that the pattern of discordance was seemingly stochastic, which led the authors to describe this phenomenon as ‘epigenetic drift’. Moreover, this study showed that the degree of discordance in DNAm patterns was also a function of the time in which the twins had been living apart, with greater discordance observed for twin pairs that had lived separate lives for longer, supporting the view that differential exposure to environmental factors drives inter-individual differences in DNAm. Soon after, Illumina produced the first of four versions of the human methylation infinium beadarray platform, allowing assessment of DNAm at approximately 1500 CpGs [21], which led to the discovery of a small number of age-associated differentially methylated cytosines (age-DMCs) [22]. Using a scaled up version of the Illumina beadarray profiling approximately 27 000 CpGs [23], subsequent studies demonstrated that age-DMCs were present in many different tissue-types and that a significant proportion of these were shared between tissue and cell-types [24,25], as well as between human and mouse [26]. Although the CpG representation on these early beadarrays was strongly biased to gene promoters, careful statistical analysis already indicated that specific sites marked by the polycomb-repressive-complex-2 (PRC2) in stem cells were preferentially enriched among hypermethylated age-DMCs, and that taking an average DNAm over these sites yielded correlative predictors of chronological age independently of cell and tissue-type [24]. Of note, all of these findings were replicated with the scaled up 450k and EPIC Illumina beadarrays profiling approximately 450 000 and 850 000 CpGs, respectively [27–30]. In effect, while individual CpG loci can display differential age-associations depending on tissue and cell type, the average DNAm over the sites in a given sample is a robust correlate of age, valid across tissue types, a key feature that can be easily recapitulated with simple stochastic simulation models (figure 1*a*). Hence, the landscape of age-DMCs that emerged from these studies is one where: (i) specific sites in the genome are more prone to acquire uni-directional DNAm changes with age; (ii) that this process of DNAm change accrual is largely shared between tissue and cell types; and (iii) that once restricted to these sites the process appears inherently stochastic. Thus, the term ‘quasi-stochastic’ is appropriate to describe these stochastically acquired DNAm changes with age, with a differential propensity that depends on genome position.

## 3. Locus-specific probabilities to acquire age-associated DNA methylation changes

The discovery that age-DMCs are highly reproducible and shared between tissue and cell types led Steve Horvath to develop his famous multi-tissue ‘epigenetic clock’ [30,32–34], a linear multivariate predictor for chronological age. Other clocks more specific to blood tissue were also developed [35,36] but were not as extensively validated. The Horvath clock displays a remarkably high degree of accuracy, with a median absolute error of only ±3 to 5 years. This accuracy is indeed remarkable given that individual loci only exhibit rather moderate effect sizes: for instance, a Horvath clock CpG in whole blood may typically only undergo at most a 10–20% DNAm change over a 50-year period, with the great majority of reproducible age-DMCs displaying only 1–5% changes. It is worth pointing out that these effect sizes are also typical for purified cell populations (figure 1*b*). This indicates that only a relatively small fraction of cells acquire age-associated DNAm changes at any given locus, and that this fraction is fairly consistent between individuals of the same age. Thus, while in single cells, the DNAm level of any given locus follows a stochastic Bernoulli process, the average DNAm measured over a cell population displays a high level of reproducibility, with a fairly large fraction of loci displaying the same directionality of change irrespective of tissue and cell type (figure 1*c*). This is not dissimilar of many other natural phenomena that are intrinsically stochastic on the smallest scales (or when sample size is small, e.g. a single cell), but which give rise to effectively deterministic behaviour on a large-scale (or when sample size is large, e.g. a cell population). Thus, an appropriate model for age-associated DNAm changes is one where individual loci in single cells have locus-specific probabilities of acquiring a DNAm change per unit time: this model can perfectly adequately explain the small observed effect sizes in a cell population and the high predictability of epigenetic clocks.

Of note, that the probability of acquiring a DNAm change is locus specific was clearly demonstrated in a study by Nejman *et al.* [37]. Nejman *et al.* focused on CpGs mapping to gene promoters that were constitutively unmethylated across many different fetal tissue-types, thus defining an appropriate ground state. The authors then ranked these CpGs by the level of DNA hypermethylation in a given normal adult tissue type, e.g. colon. Remarkably, this ranking was congruent between different normal adult tissue types, clearly indicating that the probability of acquiring age-associated DNA hypermethylation is locus specific. Moreover, the direction of DNAm change is largely determined by the methylation level in the ground state, so that unmethylated and methylated sites tend to gain and lose DNAm with age, respectively. In this way, the effectively binary DNAm landscape in the ground state is gradually eroded with age, with ensuing DNAm values being distributed more uniformly on the (0,1) interval, reflecting an increased epigenetic or DNAm entropy over the genome. Of note, the gradual trend for unmethylated and methylated CpGs to attain more intermediate DNAm values centred around 0.5 also reflects an increased DNAm entropy as defined over the cell population, as indeed a DNAm value of 0.5 can signal maximum uncertainty as to which specific single cells are methylated or unmethylated [35,38]. That age-associated DNAm changes are acquired in a locus-dependent manner was further demonstrated by a study [31], which showed that most of these 353 Horvath-clock CpGs display different probabilities of acquiring DNAm changes across tissue types (figure 1*d*) [31]. For instance, Horvath clock CpGs mapping to *ELOVL2*, *FZD8* and *GRIA2*, define age-DMCs valid across at least 10 different cell/tissue types, but the majority (approx. 80%) of the 353 CpGs defined age-DMCs only across at least three cell/tissue types. Despite this differential propensity to acquire DNAm changes, it is worth noting that most of the CpGs in the human genome display non-negligible probabilities to undergo such alterations and hence that many distinct but equally accurate epigenetic clocks for chronological age can be built [39].

## 4. Quasi-stochastic DNA methylation changes in normal cells are associated with cancer risk

Age is one of the main cancer risk factors [40]. One reason for this is that chronological age captures the cumulative effect of exposures to other cancer risk factors such as UV-light, smoking and chronic inflammation. Thus, it is conceivable that a proportion of age-associated DNAm changes measured in a given tissue type are the result of sustained lifelong exposures to environmental factors. An interesting observation, however, is that normal cells accrue DNAm changes with exposure to environmental factors, including cancer risk factors, in a manner that is also age-independent [41]. For instance, DNAm changes derived by comparing normal tissue of exposed individuals to the normal tissue of age-matched unexposed individuals, have been observed in association with smoking [42–45], inflammation [46,47], sunlight exposure [48], *Helicobacter pylori* infection [49,50], human papilloma virus (HPV) infection [51], obesity [52] and alcohol consumption [22,53,54], and interestingly, these CpGs are universally enriched for PRC2-marked sites and display strong overlap between cancer risk factors [41], a key observation we will return to in the next section.

**Figure 2.**
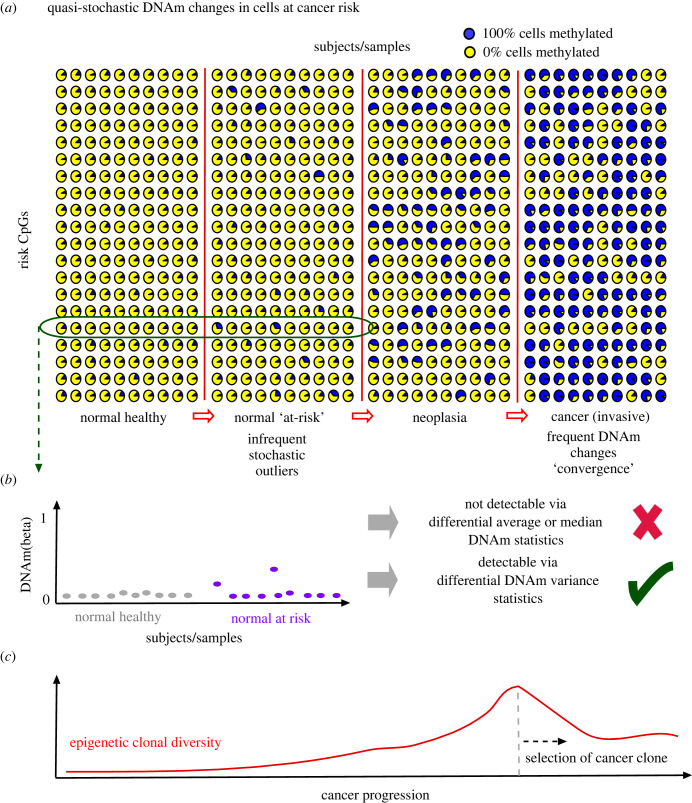
Quasi-stochastic DNAm changes in normal cells at cancer risk. (*a*) A model for how DNAm patterns across subjects and risk-CpGs change during cancer progression, including normal healthy state, normal at risk, neoplasia and invasive cancer. In this depiction, we assume all risk-CpGs are generally unmethylated in the normal healthy state. In the at-risk state, the pattern of DNAm change is inherently stochastic with individual CpGs only displaying jumps in DNAm in specific subjects. With cancer progression the patterns converge with many CpGs displaying more frequent and greater DNAm changes across many subjects. (*b*) Statistically, identifying the cancer risk-CpGs by comparing DNAm values between the normal healthy and normal at-risk states is very challenging since individual CpGs are only altered very infrequently and the alterations may not be of large magnitude. Statistics based on the differential means paradigm fail to discover the risk-CpGs. However, adopting a different feature selection paradigm based on differential DNAm variance can successfully detect such risk-CpGs. (*c*) Prediction as to how the epigenetic clonal diversity changes as a function of cancer progression, with the epigenetic diversity (this can be approximated by the inter-CpG correlations as evaluated over subjects belonging to the same disease stage) reaching a maximum immediately before the onset of invasive cancer. This pattern has been observed in real data and is consistent with the emergence of subclonal mosaicism before cancer formation and the subsequent selection of an advantageous clone (the cancer clone) at cancer onset.

Evidence of the quasi-stochastic nature of DNAm changes in relation to cancer risk factors has come from studies profiling DNAm at the earliest stages of the carcinogenic process (figure 2*a*). One of the first studies to demonstrate this was a prospective case-control study nested within a large randomized clinical trial (ARTISTIC) [55] to assess the impact of liquid-based cytology in addition to HPV-screening for predicting cervical cancer (CC) risk. The nested study profiled DNAm using Illumina beadarrays in a total of 152 women, all healthy (i.e. cytologically normal smears) at sample draw, but with half of the women developing a high-grade cervical intraepithelial neoplasia (CIN2+) three years after sample draw [51]. Women in each arm were matched for age and HPV status. Thus, the cytologically normal samples of the women who developed CIN2+ three years later can be viewed as representing normal cells ‘at cancer risk’. Consistent with the stochastic nature of DNAm alterations in such samples, statistical tests aimed at identifying CpGs with significantly different average DNAm levels between the prospective cases and controls, failed to do so (figure 2*b*). To clarify this point, a CpG that displays a significantly different average DNAm in the prospective cases would do so only if the DNAm change is seen in a significant number of these cases. Thus, the absence of genome-wide significance in finding such DMCs points towards a stochastic DNAm pattern where individual CpGs undergo cancer risk-associated DNAm changes in only a very small proportion of the prospective cases, with different CpGs displaying such alterations in different women. To demonstrate that this is indeed the case, it was proposed to identify such putative risk-CpGs by adopting a conceptual paradigm shift in feature selection, using a statistical test of differential DNAm variance that improves the sensitivity to detect the infrequent DNAm changes among prospective cases, defining differentially variable CpGs (DVCs) (figure 2*b*) [51,56]. A statistical algorithm called EVORA (epigenetic variable outliers for cancer risk prediction analysis) was developed to identify such cancer risk DVCs and to allow prospective cancer risk prediction [51,57,58]. Three key observations from the EVORA study were: (i) that the normal samples ‘at cancer risk’ displayed significantly more frequent and variable changes in DNAm compared to the normal samples that remained healthy; (ii) that the CpGs undergoing these variable DNAm changes in the normal samples at risk were not randomly distributed, instead displaying a particular preference for PRC2/bivalently marked sites in stem-cells; and (iii) that a proportion of these DNAm changes defined ‘outliers’ characterized by 20–30% ‘jumps’ in DNAm. In fact, a total of approximately 144 cancer risk loci were identified that were ultra-stably unmethylated across all normal samples, but which displayed 20–30% increases in DNAm in a very small proportion of prospective cases. These 20–30% jumps in DNAm probably reflect selection of specific subclones, with the specific CpGs undergoing these DNAm increases varying ‘stochastically’ from one prospective case to the next. Importantly, cancer risk-CpGs displayed more frequent and higher magnitude DNAm changes in high-grade cervical intraepithelial (CIN2+) lesions, with the frequency and magnitude of the DNA changes increasing further in invasive CC [51]. Thus, the initial stochastic pattern of DNAm change in normal samples ‘at-risk’ gives way to a more deterministic, convergent, yet still variable pattern of DNAm change in CIN2+ and invasive CC, where at a given locus, a DNAm change is observed for a much higher proportion of cases (figure 2*a*). Thus, in CIN2+/CC these sites are identifiable via the usual feature selection paradigm based on testing for a difference in average DNAm. In this regard it is worth noting that although a number of studies have suggested inherently stochastic and increased DNAm variation in invasive cancer [59,60], the reality is that many CpGs do display DNAm changes across a larger proportion of tumours, suggesting a less stochastic pattern compared to precursor lesions. In line with this, the dynamic patterns of DNAm change during carcinogenesis were subsequently studied in terms of epigenetic diversity, loosely defined by the magnitude of inter-CpG covariances, indicating that epigenetic clonal diversity may be maximal in the stage immediately prior to the onset of invasive cancer (figure 2*c*) [61,62]. Of note, the reduction in relative clonal diversity in invasive cancer is entirely expected given the selection of a cancer clone that defines cancer onset.

These findings of quasi-stochastic DNAm changes in normal cells at risk of developing CIN2+ have also been observed in other cancer types. For instance, a study comparing DNAm profiles of normal breast tissue from healthy women to those obtained in age-matched histologically normal tissue adjacent to breast cancer, also failed to detect cancer risk DMCs using conventional statistical methods that test for significance of differential means, but was able to detect and validate cancer risk loci by adopting the EVORA-feature selection paradigm [63]. As with the study in CC, the stochastic DNAm outliers were largely exclusive to the normal-adjacent ‘at risk’ tissue, with these same loci displaying more frequent ‘convergent’ and higher magnitude DNAm changes in the matched invasive breast cancer. Importantly, EVORA could discriminate normal healthy breast tissue from pre-invasive ductal carcinoma *in situ* with an area under the curve greater than 0.8 [63], a high discrimination accuracy not yet obtainable using alternative molecular changes such as copy number variations [64]. That DNAm outliers in normal tissue may define cancer risk markers, and their increased predictive relevance over somatic mutations, was further demonstrated in the context of oesophageal and gastric cancer [65]. Thus, given the upcoming pre-cancer genome atlas projects [66], adopting this differential variance paradigm could be critically important to develop more accurate risk prediction tools.

Given that age itself is a major cancer risk factor, one would expect age-associated DNAm changes to also display an increased stochasticity with chronological age. This has indeed been demonstrated but only in the context of blood tissue [38,67]. While most of these age differentially variable cytosines (age-DVCs) also constitute age-DMCs, they are generally speaking not part of epigenetic clock predictors, precisely because the increased variability/stochasticity with age reduces predictive accuracy. This increased stochasticity with older age however probably represents selection of subclones, as indeed heamatopoietic clonal mosaicism has been well demonstrated and is known to increase with age [68,69]. More generally, age-DVCs may represent improved markers of biological age [38], yet this awaits confirmation from future studies.

## 5. Cell division is a major driver of age-associated DNA methylation changes

**Figure 3.**
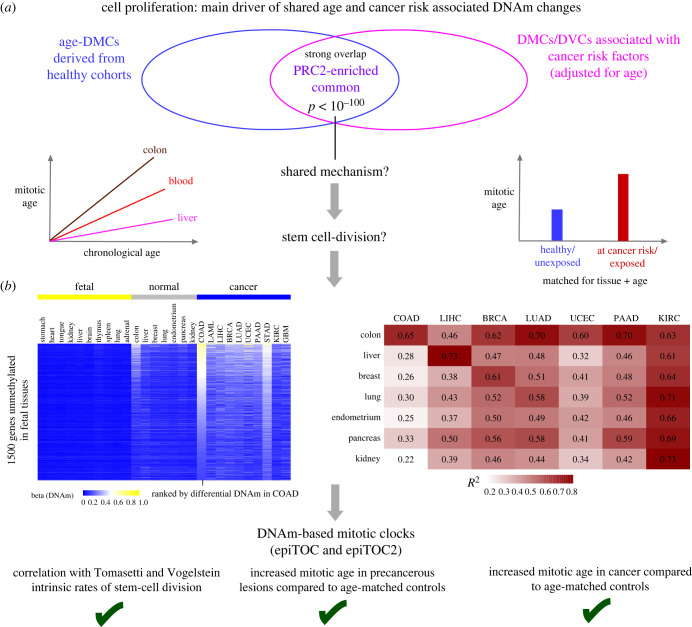
Cell proliferation: main driver of shared age and cancer risk associated DNAm changes. (*a*) Venn diagram illustrating the empirical observation that age-associated differentially methylated cytosines (age-DMCs) derived from a cohort of healthy subjects overlaps very strongly with DMCs and differentially variable CpGs (DVCs) derived by comparing healthy/unexposed to ‘at-cancer risk’/exposed subjects. This strong overlap points towards a shared mechanism, which is likely to be cell division of the stem cells in the tissue. Indeed, mitotic age would correlate with chronological with a slope dependent on the degree of cellular turnover, and mitotic age would also increase in at-risk/exposed subjects, even after adjustment for tissue and chronological age. (*b*) Heatmaps provide evidence of cell division being a driver of DNAm changes. Left heatmap compares DNAm values of constitutively unmethylated gene promoters across fetal tissue types in adult normal tissue types and corresponding cancer types, as shown. Genes promoters have been ranked according to the level of DNA hypermethylation in colorectal adenocarcinoma (COAD). Observe how the ranking is congruent with that of other cancer-types, as well as in normal colon tissue. Right heatmap displays *R*^2^ values between the DNAm profiles of normal and cancer tissues, highlighting the strong correlation between normal and its respective cancer tissue. LIHC, liver hepatocellular carcinoma; BRCK, breast cancer; LUAD, lung adenocarcinoma; UCEC, uterine cervix endometrial cancer; PAAD, pancreatic adenocarcinoma; KIRC, kidney renal cell carcinoma. This and other evidence discussed in text led to the development of DNAm-based mitotic clocks that yield proxies for the mitotic age of samples and that can predict the status of precancerous and cancer lesions.

As remarked earlier, an intriguing insight is that CpGs undergoing age-independent DNAm changes with exposure to cancer risk factors, strongly overlap with those undergoing age-associated DNAm changes in healthy unexposed individuals (figure 3*a*) [41]. Moreover, it is consistently observed that PRC2 and bivalently marked sites are strongly enriched among these sites displaying shared age and risk factor-specific DNAm changes [24,41,51]. As we shall argue below, DNAm changes associated with DNAm maintenance errors following cell division provides a likely mechanistic explanation for this overlap, since tissue-turnover correlates with chronological age (true for all tissue types except post-mitotic ones), and because the rate of tissue turnover can be increased by cancer risk factors such as smoking and inflammation (figure 3*a*).

While DNAm changes common to ageing and other cancer risk factors exist, it is also abundantly clear that cancer risk factors like smoking or obesity are associated with their own unique DNAm fingerprints [43,52]. For instance, the repressor of the aryl hydrocarbon receptor, denoted *AHRR*, is one of the top-ranked loci undergoing hypomethylation in the blood of smokers, yet this locus does not change in relation to obesity. Conversely, sites associated with obesity generally do not change in relation to smoking. Thus, it is clear that many DNAm changes associated with cancer risk factors arise through non-stochastic mechanisms that are fairly unique to the specific exposures, for instance, *AHRR* hypomethylation in the blood of smokers probably reflects an increased induction of xenobiotic metabolism enzymes. This then begs the question what mechanism(s) underpin the DNAm signatures that are shared between cancer risk factors including age? Substantial evidence supports the view that DNAm errors arising during cell division are a main driver of the shared DNAm changes across cancer risk factors (figure 3*a*). One of the first studies pointing towards cell division being a major driver of DNAm changes came from an analysis of DNAm in endometrial glands from women before and after menopause [70]. It was shown how DNAm changes accrue in the stem cells of the glands as a linear function of age up until the age of menopause, at which point DNAm levels stabilized. It is well known that cell division in endometrial glands is fuelled by oestrogen exposure, hence the observed stabilization after menopause is consistent with a lack of oestrogen and stem-cell division. Further evidence that cell division plays a central role in driving DNAm changes came from Nejman *et al.* [37] and a follow-up study [71]. By focusing on CpGs mapping to unmethylated promoters across fetal tissue types, Nejman *et al.* observed that the degree of age-associated hypermethylation at these sites was strongest for tissues like colon or stomach that have a high turnover rate (figure 3*b*). The authors further observed that the ranking of the age-associated hypermethylation changes was similar between tissue types, suggesting that a common tissue-independent process is driving these DNAm changes. Moreover, when comparing the degree of DNA hypermethylation between normal and the corresponding cancer tissue, it was observed that the ranking of the CpGs is highly robust, the only difference being the degree of DNA hypermethylation, which was much stronger in the cancer tissue (figure 3*b*). A follow-up study quantified this and showed that approximately 60% of the aberrant DNAm landscape of one cancer type (e.g. colon cancer) is explained by that of another unrelated tumour type (e.g. breast cancer) [72], once again suggesting that a common biological process underlies these DNAm changes across normal and cancer tissues. Another study compared DNAm patterns of the highly proliferative luminal-B breast cancer subtype to the less proliferative luminal-A subtype and normal tissue, finding that many gene promoters unmethylated in normal breast tissue that undergo significant DNA hypermethylation in luminal-A breast cancer, do so also in the luminal-B subtype, but that the degree of DNAm change, as well as the corresponding change in gene expression, is much bigger in the more proliferative luminal-B subtype [73]. These replication-linked DNAm patterns across breast cancer subtypes were also observed for sites undergoing hypomethylation [74]. In summary, all these patterns of DNAm change are entirely consistent with those that would arise owing to cell division, since an increased proliferation rate is a common cancer hallmark, and since it is variable between cancer types as well as between normal tissue types.

## 6. Epigenetic mitotic clocks and cancer risk

The connection between DNAm change accrual and the cumulative number of divisions in the stem-cell pool of a tissue has important implications for predicting cancer risk. Indeed, the total number of stem-cell divisions (also known as mitotic age) has been proposed to be a major cancer risk factor [75], and recent experimental mouse data obtained in multiple organs supports the hypothesis that an increased mitotic age increases cancer risk [76]. Hence, a number of recent studies developed DNAm-based clocks that yield proxies for mitotic age [77–81] (figure 3*c*). Two of these mitotic clocks, epiTOC [77] and epiTOC2 [78], are built from stochastically acquired DNAm changes at CpGs mapping to unmethylated promoters in fetal tissue, which are strongly enriched for PRC2-sites. EpiTOC2 in particular uses an explicit mathematical model of DNAm transmission following cell division that allows the intrinsic rate of stem-cell division of tissues to be estimated. These estimates were shown to correlate well with experimentally derived ones as derived and curated by Tomasetti & Vogelstein [75]. EpiTOC/epiTOC2 mitotic age estimates were also increased in normal cells at cancer risk as well as in cancer itself. Other studies have shown that DNAm-based mitotic clocks can also be built focusing on CpGs that are methylated in normal tissue but which lose methylation during cell division as a result of incomplete DNAm maintenance, these sites preferentially mapping to isolated CpGs occurring in a WCGW (W = A/T) context (termed ‘solo-CpGs’) [79,80]. Many of these solo-CpGs map to partially methylated domains (PMDs) in late-replicating regions, thus explaining their higher propensity to lose methylation following cell division [82], specially under high replicative stress conditions such as early development and cancer. Conversely, regions that replicate early and which are generally enriched for euchromatin and active or poised regions are more likely to gain methylation [82]. In summary, what these studies demonstrate is that: (i) the DNAm changes associated with mitotic age preferentially occur at certain CpGs (poised PRC2-sites for hypermethylation, PMD-late replicating sites for hypomethylation); (ii) that once restricted to these sites, DNAm accrues at these CpGs in a stochastic manner; and (iii) that average DNAm levels over these sites provide reasonably good proxies for mitotic age.

## 7. A causal epigenetic mechanism underlying cancer risk

**Figure 4.**
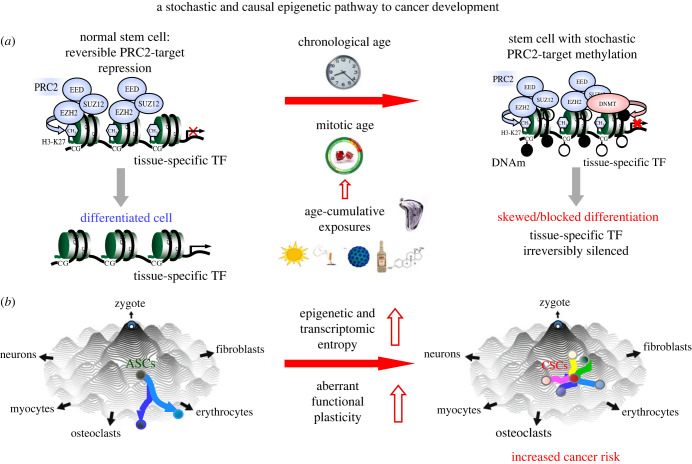
A stochastic and causal epigenetic pathway to cancer development. (*a*) Depicted is a random PRC2-marked locus associated with a tissue-specific TF that undergoes gradual stochastic age-associated DNA hypermethylation. This DNAm accrual occurs because of passage of time, cell division and exposure to cancer risk factors that may aggravate the rate of stem-cell division. The previous reversible repression of the tissue-specific TF in the adult normal stem cell, thus permissive of differentiation, is replaced by an irreversible suppression of the TF leading to a block or skew in differentiation. (*b*) Waddington epigenetic landscape depiction of this process, depicting how the adult stem cell (ASC) can no longer differentiate into downstream functional lineages, instead being forced to explore alternative routes, increasing epigenetic diversity, entropy and aberrant functional plasticity. The likelihood of this precursor cancer stem cell (CSC) then accessing an aberrant malignant state increases.

DNA hypermethylation associated with age and other cancer risk factors is enriched for PRC2 and bivalently marked sites in stem cells. An important additional observation is that many of these sites co-localize to the promoters of tissue-specific TFs [83], which are generally unmethylated in the corresponding tissue types. Hence, given the inverse association between promoter DNAm and gene expression, it is plausible that gradual DNA hypermethylation of these regulatory regions could lead to irreversible silencing of the tissue-specific TFs, which in turn could result in blocks or skews in cellular differentiation, as often observed in cancer development (figure 4*a*) [84–86]. For instance, lung, oesophageal and gastric cancer progress through well-defined consecutive stages that include dysplasias or metaplasias. Thus, although the DNAm changes may be acquired in a largely stochastic fashion, occasionally they may ‘hit’ key tissue-specific TFs, leading to blocks or skews in differentiation that may promote carcinogenic transformation. Indeed, it is worth noting that tissue-specific TFs display preferential downregulation in the corresponding cancer types [87], suggesting that this downregulation is an important feature of the cancer cell undergoing positive selection. Importantly, this inactivation of tissue-specific TFs has also been observed at single-cell resolution using single cell RNA-Seq (scRNA-Seq) data and in precursor cancer lesions [88,89]. For instance, a recent scRNA-Seq study of multi-stage oesophageal cancer development used a highly validated single-cell measure of ‘dedifferentiation’ based on the concept of diffusion entropy [89–91], to demonstrate that more stem-like cells in preneoplastic cell populations were characterized by a higher oesophageal-specific TF ‘inactivation load’, defined as the number of oesophageal-specific TFs displaying reduced differentiation activity in a cell [89]. These stem-like cells were also more likely to be selected for during oesophageal cancer progression [89]. This is entirely consistent with the cancer stem-cell hypothesis in the sense that both the cancer stem cell and the normal adult stem cell express these tissue-specific TFs at very low levels [89], and hence their observed downregulation during carcinogenesis may only reflect the selection of a stem-like state. However, the most frequent molecular change associated with their observed downregulation is promoter hypermethylation and not copy number deletions or somatic mutations [72,87,89]. This supports the view that these tissue-specific TFs are irreversibly silenced in the cancer stem cell via promoter hypermethylation, while in the adult stem cell they are expressed at low levels owing to a repressive but reversible chromatin state (figure 4*a*). Hence, it is plausible that aberrant promoter hypermethylation-induced silencing of key tissue-specific TFs could propel the adult stem cell in the tissue into a more fetal-like state endowed with an increased functional plasticity that allows these cells to acquire malignant cell-states not otherwise accessible (figure 4*b*). In line with this, several authors have proposed that an increased epigenetic and functional plasticity is a major cancer risk factor itself, as such plasticity promotes clonal diversity, which in turn naturally increases the probability of a new cancer-promoting mutation (or epimutation) establishing itself in the cell population [92,93]. Alternatively, such cancer driver mutations may pre-exist in the normal ageing cell population but only become effective once the adult stem cells occupy the more plastic aberrant states [94–97]. While not directly relevant to the stages preceding cancer development, it is worth pointing out several studies demonstrating how epigenetic clonal diversity at the DNAm level is predictive of cancer progression and clinical outcome [98–100]. For instance, one study identified promoter hypermethylation cancer driver events associated with poor clinical outcome in chronic lymphocytic leukaemia, and obtained experimental proof via CRISPR/Cas9 knockouts that inactivation of the corresponding tumour suppressor genes provides a fitness advantage [101]. Based on these findings, it would be interesting to explore the effect of such knockouts in normal cells and whether this leads to an increased epigenetic clonal heterogeneity.

While the above model of oncogenesis hinges on age-associated DNAm changes affecting tissue-specific TF expression, it is worth remembering that such deregulation of TFs could occur by other means. Indeed, one of the most important findings in cancer genomics has been the frequent identification of somatic mutations in epigenetic regulators (e.g. DNMT3A, TET2, ARID1A, IDH1/2, EZH2) as key cancer drivers [102,103]. For some cancer types (e.g. leukaemias), these somatic mutations have been shown to increase in frequency with age in line with the observed increased clonal mosaicism [104,105], potentially inducing widespread genomic DNAm changes that may result in deregulation of tissue-specific TFs. As a concrete example, DNMT3A loss has been shown to impair proper heamatopoietic stem-cell differentiation promoting a skew towards myeloid differentiation [106].

## 8. Conclusion

Here I have advanced the thesis that stochastically acquired DNAm changes that accrue in the adult stem-cell pool of tissues as a result of tissue turnover, may define a causal epigenetic path to an elevated cancer risk. Such DNAm changes, acquired during cell division, would naturally correlate with both chronological age, as well as with sustained exposure to cancer risk factors that accelerate the intrinsic rate of stem-cell division. This simple model provides a mechanistic understanding as to why different cancer risk factors may share a common DNAm signature. This common DNAm signature is enriched for PRC2-marked gene promoters that are unmethylated in fetal tissue, pointing towards a quasi-stochastic process of aberrant DNAm acquisition that can yield clinically valuable proxies for mitotic age. Given that these sites are also enriched for tissue-specific TFs, this model provides a causal pathway to cancer development, mediated by irreversible silencing of these TFs, which leads to an increased epigenetic entropy and aberrant functional plasticity, and ultimately to an increased cancer risk.

## Data Availability

Data availability statement is included in the main article file. Data displayed in figure 1*b* derives from Illumina 450k DNAm data generated by Reynolds *et al.* [107]. figure 1*c* derives from Illumina 450k DNAm data (BLUEPRINT) [108], previously published by us in Zhu *et al.* [31]. Data displayed in figure 1*d* derives from Illumina 450k DNAm data analysed by us in Zhu *et al.* [31]. Data panels from figure 3*b* are taken from our previously published work Chen *et al.* [71].
